# C-954-01. Alcohol Dependence Before Burn Injury: An Intoxicating Driver of Long-term Psychiatric Morbidities and Mortality

**DOI:** 10.1093/jbcr/irag033.133

**Published:** 2026-04-06

**Authors:** Sarah Wang, Matthew Q Dao, Paul Won, Erin Wolfe, Karel-Bart Celie, Sarah A Stoycos, Haig A Yenikomshian

**Affiliations:** Keck School of Medicine, University of Southern California, Los Angeles, CA; University of Texas Medical Branch - John Sealy School of Medicine, Galveston, TX; Division of Plastic Surgery, Department of Surgery, McGovern Medical School, University of Texas Health Science Center at Houston, Houston, TX; Division of Plastic and Reconstructive Surgery, Keck School of Medicine, University of Southern California, Los Angeles, CA; Division of Plastic and Reconstructive Surgery, Keck School of Medicine, University of Southern California, Los Angeles, CA; Keck School of Medicine, University of Southern California, Los Angeles, CA; Division of Plastic and Reconstructive Surgery, Keck School of Medicine, University of Southern California, Los Angeles, CA

## Abstract

**Introduction:**

Alcohol use disorder is prevalent among burn patients, with studies estimating that 30–50% present with intoxication or a history of dependence at the time of injury. Burn survivors face substantial psychological challenges that complicate recovery including elevated risk of psychiatric hospitalization, substance use relapse, and suicide attempts for years after the initial burn injury. However, the independent impact of pre-injury alcohol dependence on post-burn psychiatric outcomes has not been well delineated. The purpose of this study was to perform a nationwide analysis to evaluate the effect of alcohol dependence prior to burn injury on long-term psychiatric disorders following burn injury.

**Methods:**

A globally federated database of over 130 million de-identified health records was reviewed for adult burn patients between April 2010 and June 2025. Two cohorts were established: patients with a documented diagnosis of alcohol dependence prior to burn injury (n = 7148) and patients without alcohol dependence before or after burn injury (n = 731 761). Patients with pre-existing psychiatric comorbidities before their burn event were excluded. Both cohorts were 1:1 propensity score matched based on demographics, comorbidities (diabetes, obesity, kidney and liver diseases, and cardiovascular disease), concurrent substance use disorders (nicotine dependence, cannabis use, and cocaine addiction), burn severity (total body surface area affected, region of bodily injury, and degree), and procedural interventions (debridement and autografts). Risk ratios (RRs) were calculated to compare psychiatric disorders and readmission rates at 90 days and 12 months post-burn. Statistically significant threshold was determined as *p*<.05.

**Results:**

Following propensity score matching, each cohort consisted of 6928 patients. At 90 days, alcohol-dependent patients had significantly higher rates of readmission (RR = 1.87), opioid use disorder (RR = 1.91), depression (RR = 2.68), anxiety (RR = 2.13), post-traumatic stress disorder (RR = 2.35), adjustment disorder (RR = 1.77), suicidal ideation (RR = 2.82), and sleep disorders (RR = 1.56) (all *p*<.05). At 12 months, all associations remained significant, with the addition of increased mortality (RR = 1.27, *p*<.0001) in the alcohol-dependent group.

**Conclusions:**

Pre-injury alcohol dependence was independently associated with significantly higher risks of psychiatric morbidities, readmission, and mortality after burn injury. These findings emphasize the importance of early systematic screening for alcohol dependence in admitted burn patients, followed by treatment of alcohol dependence, to improve psychiatric recovery.

**Applicability of Research to Practice:**

Improved understanding of these associations in burn patients may help clinicians identify high-risk patients early and tailor psychiatric support to reduce the risk of psychiatric sequelae.

**Funding for the study:**

N/A.

